# SH2B1 promotes NSCLC cell proliferation through PI3K/Akt/mTOR signaling cascade

**DOI:** 10.1186/s12935-018-0632-x

**Published:** 2018-09-06

**Authors:** Shaoqiang Wang, Yingying Zheng, Zhiwei He, Wolong Zhou, Yuanda Cheng, Chunfang Zhang

**Affiliations:** 1 Department of Thoracic Surgery, Affiliated Hospital of Jining Medical University, Jining Medical University, Jining, 272029 Shandong People’s Republic of China; 2 Department of Endocrinology, Affiliated Hospital of Jining Medical University, Jining Medical University, Jining, 272029 Shandong People’s Republic of China; 3 Department of Thoracic Surgery, Xiangya Hospital, Central South University, No. 87, Xiangya Road, Changsha, 410008 Hunan People’s Republic of China

**Keywords:** SH2B1, Proliferation, AKT, NSCLC

## Abstract

**Background:**

Non-small cell lung cancer (NSCLC), the most prevalent type of human lung cancer, is characterized by many molecular abnormalities. SH2B1, a member of the SH2-domain containing family, have recently been shown to act as tumor activators in multiple cancers. The objective of this study was to investigate the role SH2B1 and the underlying molecular mechanism in NSCLC.

**Methods:**

Cell functional analysis and cell line-derived xenograft model were performed to determine SH2B1 potential roles on NSCLC cell proliferation in vitro and in vivo. In vitro assays were performed to identify signal molecular mechanisms. Subsequently, 104 patients with NSCLC undergoing primary surgical resection were recruited to evaluated expression of SH2B1 and Akt/mTOR signaling markers by immunohistochemical staining to determine their clinicopathologic significance.

**Results:**

Modulation of SH2B1 expression levels had distinct effects on cell proliferation, cell cycle and apoptosis in the NSCLC cell lines A549 and H1299. At the molecular level, overexpression of SH2B1 resulted in the upregulation of the Akt/mTOR markers, p-Akt and p-mTOR, and downregulation of PTEN to promote NSCLC cell proliferation, while silencing SH2B1 had the opposite effect. In human NSCLC specimens, SH2B1 expression levels were positively associated with Akt/mTOR signaling pathway markers.

**Conclusions:**

The SH2B1/Akt/mTOR/PTEN axis is required for regulating NSCLC cell proliferation and might prove to be a promising strategy for restraining tumor progression in NSCLC patients.

**Electronic supplementary material:**

The online version of this article (10.1186/s12935-018-0632-x) contains supplementary material, which is available to authorized users.

## Background

NSCLC is the leading cause of cancer-related deaths, with 733,300 cases being diagnosed in China in 2015 [1]. Standard treatments, involve surgical resection, chemotherapy and radiation, are rarely curative and the 5-year survival rate still remains at a dismal 15.6% in United States, and even worse in China [2]. Patients with localized disease or with distant metastasis at diagnosis have a significantly different 5-year survival rate from 52 to 3.6%, which motivates better screening methods to detect early-stage cancers [2].

NSCLC is a heterogeneous disease with diverse morphological characteristics, metastasis patterns and clinical outcomes, mainly due to be with distinct oncogenic driver events, that is different molecular signals. Thus, getting a better understanding of the molecular pathogenesis of NSCLC is a critical first step to develop effective targeted therapies to improve outcomes. The SH2B1 [Src homology 2(SH2) B adaptor protein 1] gene encodes a member of the SH2-domain containing mediators family, which facilitates and enhances catalytic activity of its bound enzymes via couple upstream activators of multiple receptor tyrosine kinases to downstream effectors [3]. In multiple malignancies including NSCLC, the up-regulation of SH2B1 is positively correlated with tumor TNM stages and poor survival rate [4]. Amplification of SH2B1 is correlated with increased cell proliferation and survival by regulating mitogenic responses [5–7] and loss of cell contact inhibition [6], which is thought to be mediated by dysregulation of various mitogenic and proliferative signaling mediators, such as PDGF-BB, IGF-1, insulin, PI3K [7] and mTORC1 [8]. Not surprisingly, in cancer cells activation of RET oncogenic signaling, which can be induced and enhanced by SH2B1, can override growth suppressor (RET inhibitors) activities and stimulate cell cycle progression and further potentiates the neoplastic transformation [9]. Therefore, understanding the mechanisms underlying proliferation induced by SH2B1 expression not only helps to understand NSCLC growth promotion, but also can provide a platform to develop a novel therapeutic option in NSCLC therapy.

In the present study, we sought to address this gap in knowledge about the potential role of SH2B1 associated with human NSCLC. Here, we demonstrate that in SH2B1-expressing NSCLC cells, SH2B1 has a functional role in promoting proliferation by modulation of mitogen-activated protein kinase signaling, phosphatidylinositol 3-kinase (PI3K)/Akt/mammalian target of Rapamycin (mTOR) signaling pathway and inducing an elevated level of the transcription factor cyclin D1. In addition, SH2B1 overexpression reduced apoptosis by activating caspase-3. These findings revealed a role of SH2B1 in the development of NSCLC.

## Materials and methods

### Ethical approval and Informed consent statements

Written informed consent was obtained from 104 participants before the study. The clinical protocol for this study was approved by the Medical Research Ethics Committee of Xiangya Hospital, Central South University (CSU) (#201403216). All mice experiments were approved by the Animal Ethics Committee and conducted by the official recommendations for the Care and Use Laboratory Animals of Xiangya Hospital, CSU (#201403217).

### Tissue specimens

A total of 104 fresh NACLC tumor specimens were archived by radical resection from those NSCLC patients without any chemotherapy and radiotherapy intervention at the Department of Thoracic Surgery of Xiangya Hospital from January 2010 to December 2011. The tumor-node-metastasis was classified according to the criteria of the 8th AJCC/UICC (American Joint Committee on Cancer/International Union Against Cancer) TNM staging system. There were 29 cases in Stage I, 33 cases in Stage II and 42 cases in Stage III. All freshly collected NSCLC tumor tissues were frozen and stored in liquid nitrogen until required. All surgical resection samples were evaluated by a board-certified pathologist.

### Cell culture

The human NSCLC cell lines A549 and H1299 (ATCC, Manassas, VA, USA) were cultured in RPMI 1640 (Gibco, Gaithersburg, VA, USA) supplemented with 10% fetal bovin serum (FBS) and penicillin/streptomycin and then incubated at 37 °C in a humidified 5% CO_2_ atmosphere.

### RNA extraction and qRT-PCR analyses

Total RNA from cultured cells was extracted using the TRIzol reagent (Invitrogen, Thermo Fisher Scientific, USA) according to the manufacturer’s manual. The expression level was quantified using SYBR Green Assay Kit (Takara, Japan). Real-time PCR was performed in triplicate using the Applied Biosystems ViiA 7 Sequence Detection system (Life technologies, CA, USA). GAPDH was used to normalize mRNA and calculate the relative expression of each transcript. The following pairs of primers were synthesized as: C-Myc (F): 5′-CCTCCACTCGGAAGGACTATC-3′, (R): 5′-TTCGCCTCTTGACATTCTCC-3′; GAPDH (F): 5′-ACCCACTCCTCCACCTTTGACG-3′, (R): 5′- TCTCTTCCTCTT GTGCTCTTG-3′.

### Immunohistochemistry (IHC)

IHC analysis was performed on 4 µm-thick formalin-fixed and paraffin-embedded NSCLC tissue sections from each tumor sample, using the PV-60001/PV-6002 Two-Step IHC Detection Reagent (ZhongShan Golden Bridge Bio, Beijing, China) according to the manufacturer’s protocol. In brief, antigen retrieval was performed in buffer for 10 min in a microwave oven. Endogenous peroxidase activity was blocked with 3% hydrogen peroxide for 20 min at room temperature. Slides were incubated with

the following primary antibodies, anti-SH2B1 (1:100, ab196575, Abcam), anti-Akt (1:500, ab8805, Abcam), anti-Akt (phospho S473) (1:200, ab8932, Abcam), anti-mTOR (1:1000, ab2732, Abcam), anti-mTOR (phosphor S2448) (1:50, ab109268, Abcam), anti-PTEN (1:100, ab31392, Abcam, Cambridge, MA, USA), anti-Ki67 antibody (1:200, Cell signaling, Danvers, MA, USA) at 4 °C overnight. Following washing with PBS, slides were incubated with HRP-labeled secondary antibody for 50 min at room temperature. Immunoreactive products were visualized with 3,3′-diaminobenzidine tetrahydrochloride (DAB) and counterstained with Mayer’s hematoxylin. For negative controls, slides were treated with 0.01 mol/L PBS instead of primary antibodies under the same conditions.

The IHC staining scores were determined by combining the intensity of staining and the proportions of positively stained cells. The standard of staining intensity was graded that: 1, no staining; 2, weak staining (light yellow); 3, moderate staining (yellow brown); and 4, strong staining (brown). The positive cell proportions were scored according to the following standard: 0, < 5% positive cell; 1, 6–25% positive cells; 2, 26–50% positive cells; 3, 51–75% positive cells; 4, 76–100% positive cells. We evaluated protein expression using staining index (SI), which was calculated of the proportion of positive cells and the staining intensity score as possible total scores of 0, 1, 2, 3, 4, 5, 6, 7, and 8. Samples with SI ≤ 4 were defined as low (negative) expression and SI > 4 as high (positive) expression. The degree of SI was reviewed and scored separately by two independent pathologists.

### Xenografted tumor model and H&E staining

Male BALB/c-nu mice (age of 4–5 weeks, 16–18 g) were purchased from the Hunan Slack King of Laboratory Animal Co., Ltd (Changsha, China). Mice were randomly divided into groups (*n *= 6 per group) before injection. A total of 1 × 10^7^ cells were subcutaneously injected into the left dorsal flank per mouse. Measure tumors length and width and then calculate tumor volumes (L × W^2^/2) with calipers on alternate days. On day 30, the mice were euthanized and tumors were excised to paraffin embedded. Serial 4.0 μm sections were cut and subjected to haematoxylin and eosin (H&E) staining and IHC analyzed using an anti-Ki67 (1:200, Cell signaling, Danvers, MA, USA). The proportion of Ki67 positive cells were counted as proliferation index. All mice experiments were approved by the Animal Ethics Committee and conducted by the official recommendations for the Care and Use Laboratory Animals of Xiangya Hospital, CSU (#201403217).

### Western blotting

Whole-cell lysates were prepared from human NSCLC cell lines after harvesting and centrifuging. Protein concentrations were quantified using BCA kit (Beyotime Bio, Shanghai, China). The proteins were denatured, electrophoresed (SDS-PAGE gel Kit, Beyotime Bio, Shanghai, China), and then transferred to PVDF membranes (Millipore Co., MA, USA). Subsequently, the PVDF membranes were blocked with non-fat milk powder, incubated with primary and secondary (HRP-labeled goat anti-rabbit IgG) antibodies, and visualized (Bio-Rad Image Lab Software).

Western blotting was performed using the primary antibodies, anti-SH2B1 (1:1000, ab196575, Abcam), anti-Akt (1:600, ab8805, Abcam), anti-Akt (phospho S473) (1:300, ab8932, Abcam), anti-mTOR (1:1500, ab2732, Abcam), anti-mTOR (phosphor S2448) (1:1000, ab109268, Abcam), anti-PTEN (1:600, ab31392, Abcam), anti-C-Myc (1:1000, ab32072, Abcam), antibodies (Abcam, Cambridge, MA, USA), and anti-GAPDH antiboty (1:3000. D110016, Sangon Biotech, Shanghai, China). Following the initial western blotting assay, the membranes were stripped and the band intensities were relative to GAPDH. The quantification of protein bands was performed using Image J software.

### Cell proliferation assay

2 × 10^3^ cells were seeded and cultured in triplicates in 96 well plates. Cell viability was measured every day over a 5-day period using cell count kit-8 (CCK8, #CK04, Dojindo Molecular Technologies, Rockville, MD 20850, USA). Cells were incubated with 10 μl CCK8 for 1 h at 37 °C, and absorbance was measured at 450 nm using the Elx808 Absorbance Microplate Reader (BioTek Instruments, USA).

### Colony formation assays

Cells were suspended in 0.6% agarose and placed in 24-well plates at low density (2.5 × 10^2^ cells per plate). After 2 weeks, the colonies were fixed with methanol and counted.

### Cell cycle analysis

Cells were trypsinized, collected and rinsed twice with PBS and fixed with cold absolute alcohol at 4 °C overnight. After fixation, cells were incubated with 0.5 ml PBS, 5 μl propidium iodide (20×) and 10 μl RNase A (50×) for 30 min at 37 °C without light before analysis. The cell cycle was analyzed by a flow cytometry (BD, San Jose, CA, USA).

### Flow cytometry detection

Cells were collected and stained using the FITC Annexin V Apoptosis Detection Kit I (BD Pharmingen, San Diego, CA, USA). Cell apoptosis were quantified using a flow cytometry (BD, San Jose, CA, USA).

### Gene knockdown and gene overexpression

shRNA against SH2B1 and control shRNA were constructed into the lentiviral vector pHY-LV-KD5.1 (GFP-lentivirus, Hanyin Co. Shanghai, China). The recombinant SH2B1-shRNA lentivirus (target sequence: 5′-TAACAACCAGTACTCCTTCGTGTGAGC-3′) and the negative control lentivirus (target sequence: 5′-TTCTCCGAACGTGTCACGT -3′) were prepared and titered to 10^9^ TU/ml (transfection unit). To obtained a stable SH2B1-knockdown cell line, 2 × 10^5^ A549 cells seeded in per well of six-well dishes. The cells were then infected with the same titer virus with 8 μg/ml polybrene on the following day. Approximately 72 h after viral infection, the culture medium was replaced with selection medium containing 4 μg/ml puromycin after GFP expression was confirmed under a fluorescence microscope. The cells were cultured for at least 14 days. The puromycin-resistant cell clones were isolated, amplified in medium containing 2 μg/ml puromycin for 7–9 days, and then transferred to a medium without puromycin. The clones were designated shSH2B1 (knock down) or shCtrl (negative control) cells.

Lentiviral vectors for human SH2B1 expression sequence were constructed by Hanyin Co. (Shanghai, China). The cDNA of SH2B1 with a C-terminal Flag-tag were cloned into the lentiviral vector pHY-LV-OE1.6. Stable cell lines expressing SH2B1 were generated by retroviral infection using H1299 cells, and selected with 2 μg/ml puromycin for 7–9 days. Further details of methods were carried out as gene knockdown described. The clones were designated H1299-SH2B1 (overexpression) or H1299-vector (negative control) cells. Transfection was conducted using Lipofectamine 2000 (Invitrogen, Grand Island, NY, USA) according to the manufacturer’s instructions [10].

### Statistical data analysis

All statistical analyses in this study were carried out using SPSS 22.0 statistical software. Data were shown as mean ± SD, and 2-tailed paired Student’s *t*-test was used for comparing the difference between groups unless otherwise stated. *P *< 0.05 was considered statistically significant.

## Results

### Functional significance of SH2B1 in NSCLC cells in vitro

**Fig. 1:**
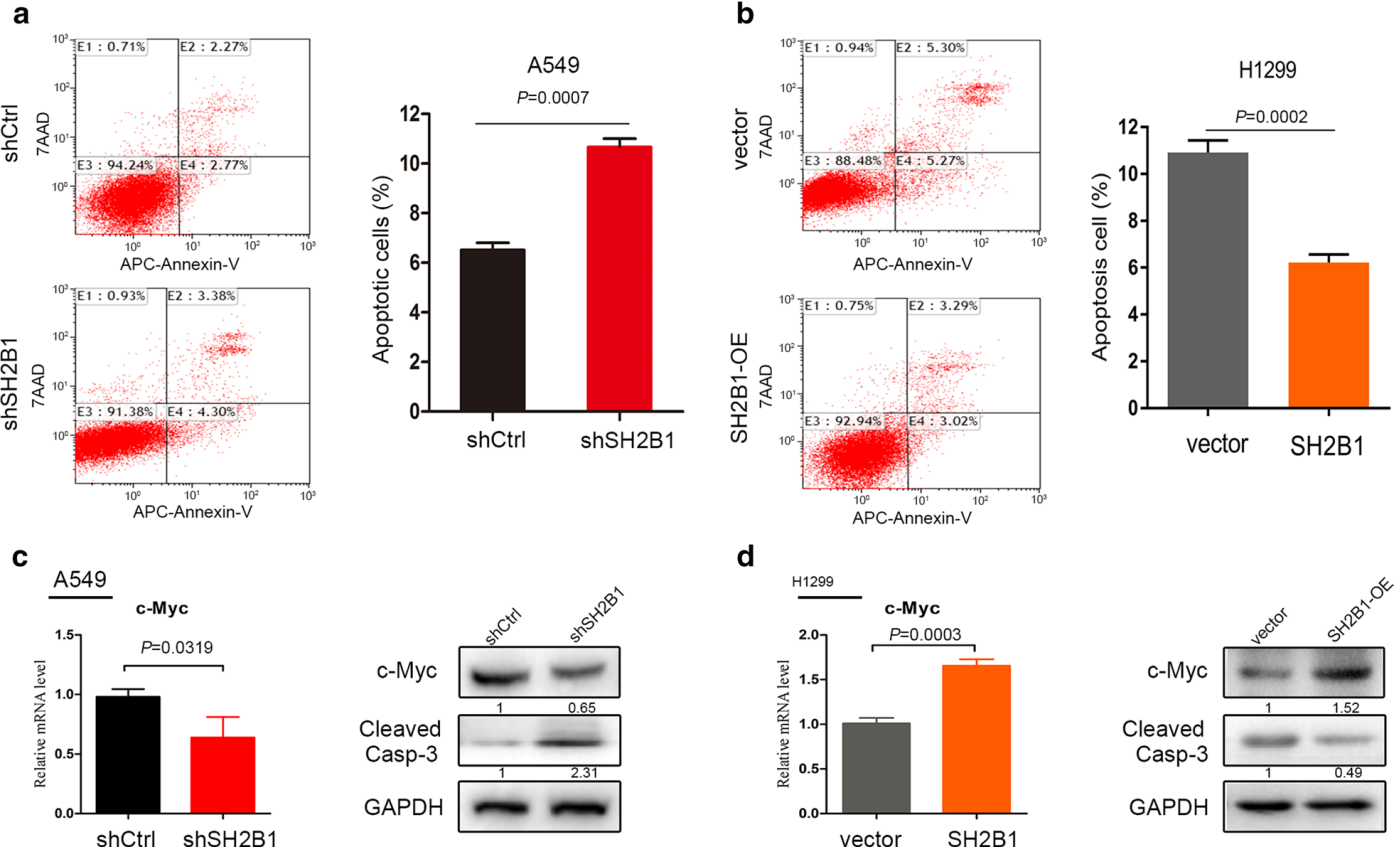
Functional significance of SH2B1 in NSCLC cells in vitro. **a**, **b** FACS analysis with annexin V-PE and 7-AAD was used to evaluate the apoptosis. The FACS-apoptosis staining (**a**) showed an increased percentage of cell apoptosis in SH2B1 knockdown A549 cells (shSH2B1) compared with control cells (shCtrl) (11.55 ± 0.68% vs 6.45 ± 0.56%, *P *= 0.0007, n = 3). **b** A decreased percentage of cell apoptosis in SH2B1 overexpression H1299 cells compared with control cells (vector) (10.91 ± 0.30% vs 6.22 ± 0.20%, *P *= 0.0002, n = 3). Results are representative of three independent experiments. **c**, **d** SH2B1 regulated the c-myc and cleaved casp-3 in A549 and H1299 cells as confirmed by qRT-PCR and western blotting analysis, mean ± SD, n = 3. The band in western blotting was normalized to GAPDH

In a previous study, we had observed that SH2B1 was significantly upregulated in NSCLC tissues and cell lines, and high SH2B1 expression was associated with tumor size [4]. We next explored its functional significance in NSCLC. Lentivirus-mediated knockdown of endogenous SH2B1 (in A549) and upregulation of SH2B1 (in H1299) were performed to evaluated the cellular outcomes using several assays [11]. To investigate whether SH2B1 affects cell cycle in NSCLC cells, we performed FACS cells cycle distribution analysis. Knockdown of SH2B1 in A549 cell lines, which had high level of SH2B1, resulted in decreased percentage of S phase (*P *= 0.0023) cells and increased cells residing at the G1 phase (*P *= 0.0002, Additional file 1: Figure S1A), reflecting that fewer A549-shSH2B1 cells were cycling and further indicating a slowed cell proliferation rate. While cyclin D1 was required for overcoming the G1/S checkpoint, so we performed the RT-qPCR and Western blot. And the data revealed that cyclin D1 in A549 cells were significantly decreased after transfection of SH2B1-shRNA compared with control shRNA (Fig. 1c). Meanwhile, flow cytometry analysis revealed that overexpression of SH2B1 had a significant shift from G1 to S phase (*P *= 0.0110, Additional file 1: Figure S1B), and this effect was associated with induction of the expression of cyclin D1 (Fig. 1d), and so accelerated the cell cycle progression.

In addition, increased cells’ G1-S phase propagation in NSCLC was linked to reduced apoptosis. To further test the impact of SH2B1 on the apoptosis in NSCLC cells, we performed an apoptosis assay by FACS analysis. Transfection of SH2B1-shRNA resulted in a significant increase in the percentage of apoptotic cells, compared with the control shRNA, in A549 cells (*P *= 0.0007, Fig. 1a). Moreover, knockdown of SH2B1 dramatically increased the level of caspase-3 activation, which play important role in apoptosis, compared with A549-shCtrl (Fig. 1c). In line with this, decreased apoptotic cells (*P *= 0.0209, Fig. 1b) and caspase-3 activation (Fig. 1d) were seen in H1299-SH2B1 cells compared with control H1299-vector.

### SH2B1 promotes NSCLC cell proliferation in vitro

**Fig. 2:**
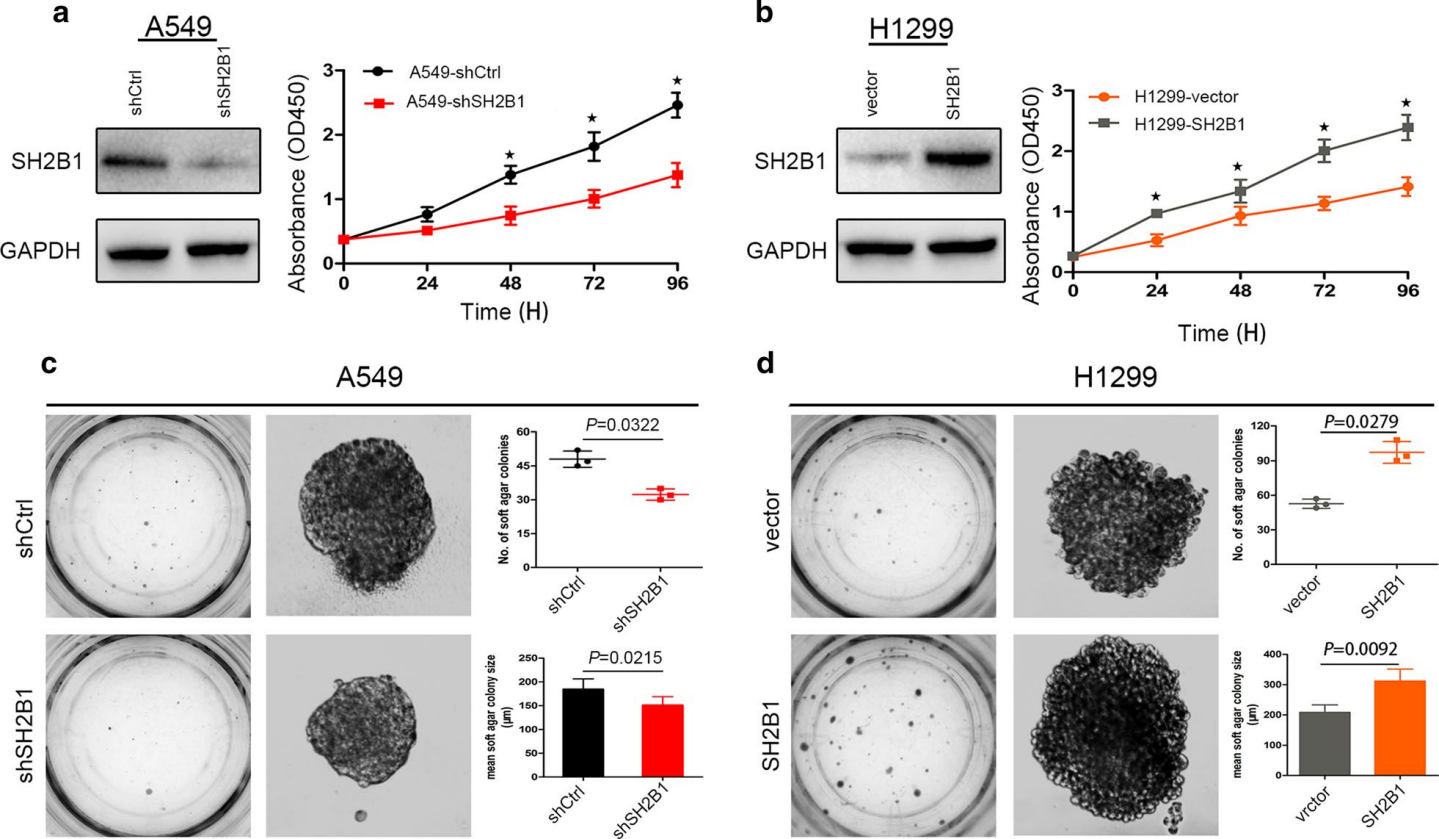
SH2B1 promotes NSCLC cell proliferation in vitro. Cell Counting Kit-8 (CCK8) and soft agar assays (number and mean size of clonogenic colonies estimated after 2 weeks) were performed to analyze A549 and H1299 cells proliferation. **a** SH2B1-knockdown suppresses the proliferation of A549 cells. **b** SH2B1 overexpression elevates the H1299 cells proliferation. *P < 0.05, n = 3. **c** Soft agar colony formation assay of SH2B1-knockdown and control A549 cells in a 24-well dish (5 × 10^2^ cells per well) for 2 weeks (n = 3). Representative images (left) and quantification of number of colonies (*P *= 0.0322, right upper) and average size of colony were shown (*P *= 0.0215, right lower). **d** Soft agar colony formation assay of SH2B1-overexpression and control H1299 cells in a 24-well dish (5 × 10^2^ cells per well) for 2 weeks (n = 3). Representative images (left) and quantification of number of colonies (*P *= 0.0279, right upper) and average size of colony were shown (*P *= 0.0092, right lower). Data were mean ± SD, n = 3

The above data indicated that SH2B1 was involved in the regulation of NSCLC cell cycle progression and cell apoptosis. We speculated that SH2B1 may play an essential role in NSCLC cell proliferation. To investigate this hypothesis, we performed CCK8 assay in A549 and H1299 cell line. Knockdown of SH2B1 in A549 cell significantly inhibited cell proliferation compared to control A549-shCtrl cells (*P *< 0.001, Fig. 2a). Similarly, the proliferation rate of H1299 cells were significantly increased after overexpression of SH2B1 and the number of H1299-SH2B1 cells was more than that of control H1299-vector cell (*P *< 0.001, Fig. 2b).

Similar results obtained from soft agar colony formation assay. SH2B1 knockdown attenuated the colony formation of A549 cells, as the number (*P *= 0.0322) and mean size (*P *= 0.0215) of single-cell-derived soft agar colonies were both markedly decreased (Fig. 2c). Moreover, over-expression of SH2B1 caused to significantly elevated colony and sphere forming capacity of H1299 cells (Fig. 2d).

### SH2B1 promotes NSCLC cell proliferation in vivo

**Fig. 3:**
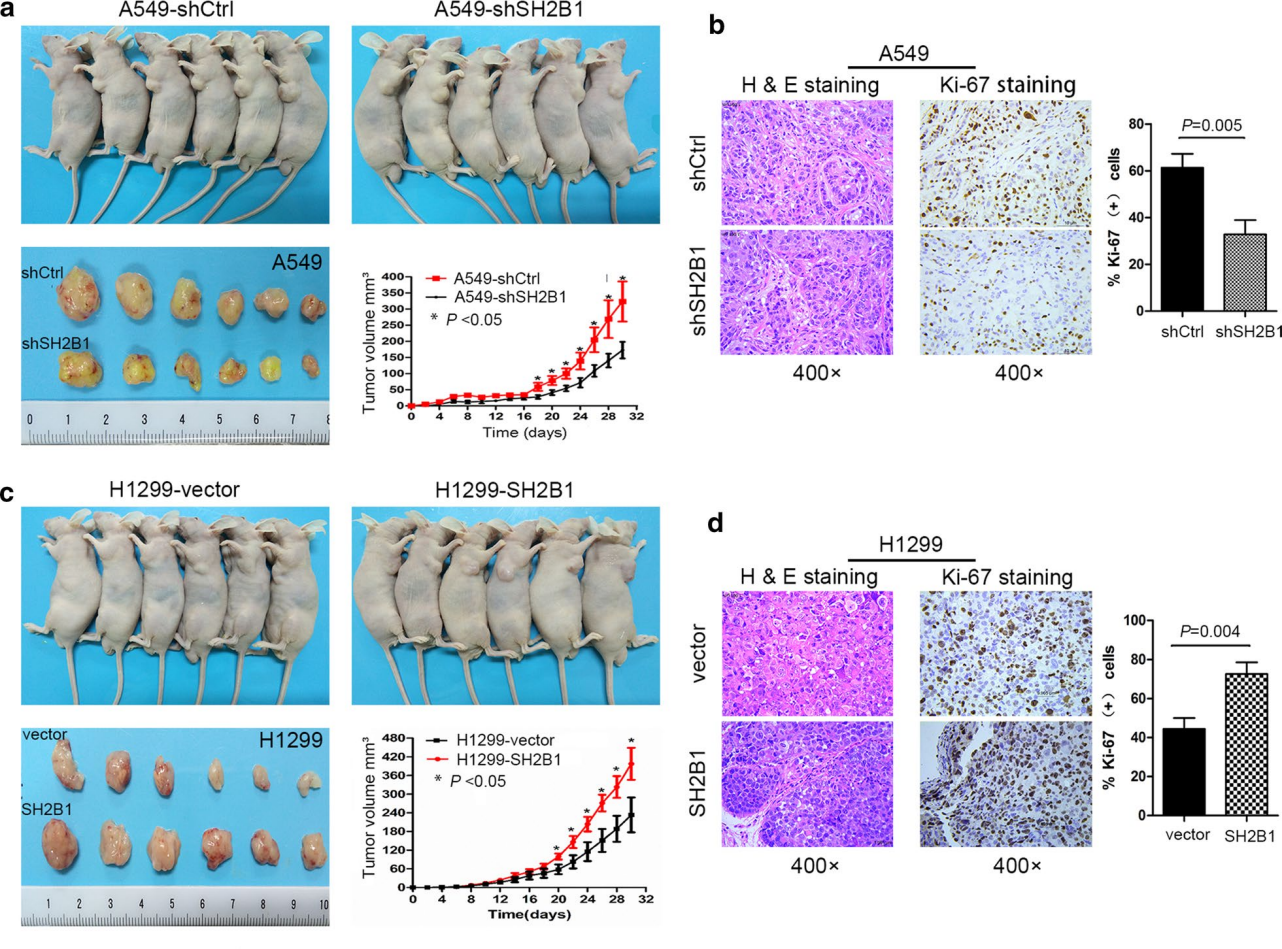
SH2B1 promotes NSCLC cell proliferation in vivo. A total of 1 × 10^7^ cells (A549 or H1299) were subcutaneously injected into the left dorsal flank per mouse. **a** SH2B1-knockdown suppressed xenograft tumor growth of A549 cells in vivo (n = 6). Upper and lower (left) panels: images of tumors from all mice in each group; lower panel (right): tumor volume (L × W^2^/2) was measured at internal day (*P < 0.05, t-test); **b** representative photographs of H&E and Ki-67 IHC staining (*P *= 0.005) in the indicated tumors. **c** SH2B1-overexpression promoted xenograft tumor growth of H1299 cells in vivo (n = 6). Upper and lower (left) panel: images of tumors from all mice in each group; lower (right) panel: tumor volume was measured at internal (*P < 0.05, t-test); **d** representative photographs of H&E and Ki-67 IHC staining (*P *= 0.004) in the indicated tumors

To verify the positive role of SH2B1 in NSCLC cells growth, in vivo nude mice (age, 4 weeks) were performed xenograft tumor assays using A549 cells stably transfected with either SH21-shRNA (abbreviated as shSH2B1 hereafter simplicity) or negative control lentiviruses (shCtrl). Subcutaneous tumor xenografts were monitored on alternate days (Fig. 3a, c). For A549 cell lines, the shSH2B1 cells grew tumors at a much slower rate compared to shCtrl cells (Fig. 3a). At the time of sacrifice, shSH2B1 cell tumors were significantly smaller compared with those of shCtrl cells (Fig. 3a). Furthermore, xenograft tumors cell proliferation was assessed by Ki-67 immunostaining. The estimated result obtained from Ki-67 staining sowed SH2B1-knockdown (shSH2B1) tumors displayed significantly less proliferative cells (Fig. 3b). Most notably, overexpression of SH2B1 in H1299 cells (H1299-SH2B1) significantly increased the tumor growth rate and tumor mass in vivo (Fig. 3c). In addition, the number of Ki-67-posive cells were high in SH2B1-overexpression tumors compared with control cells containing vector control (Fig. 3d).

### SH2B1 regulates NSCLC cell proliferation through Akt/mTOR pathway in vitro

**Fig. 4:**
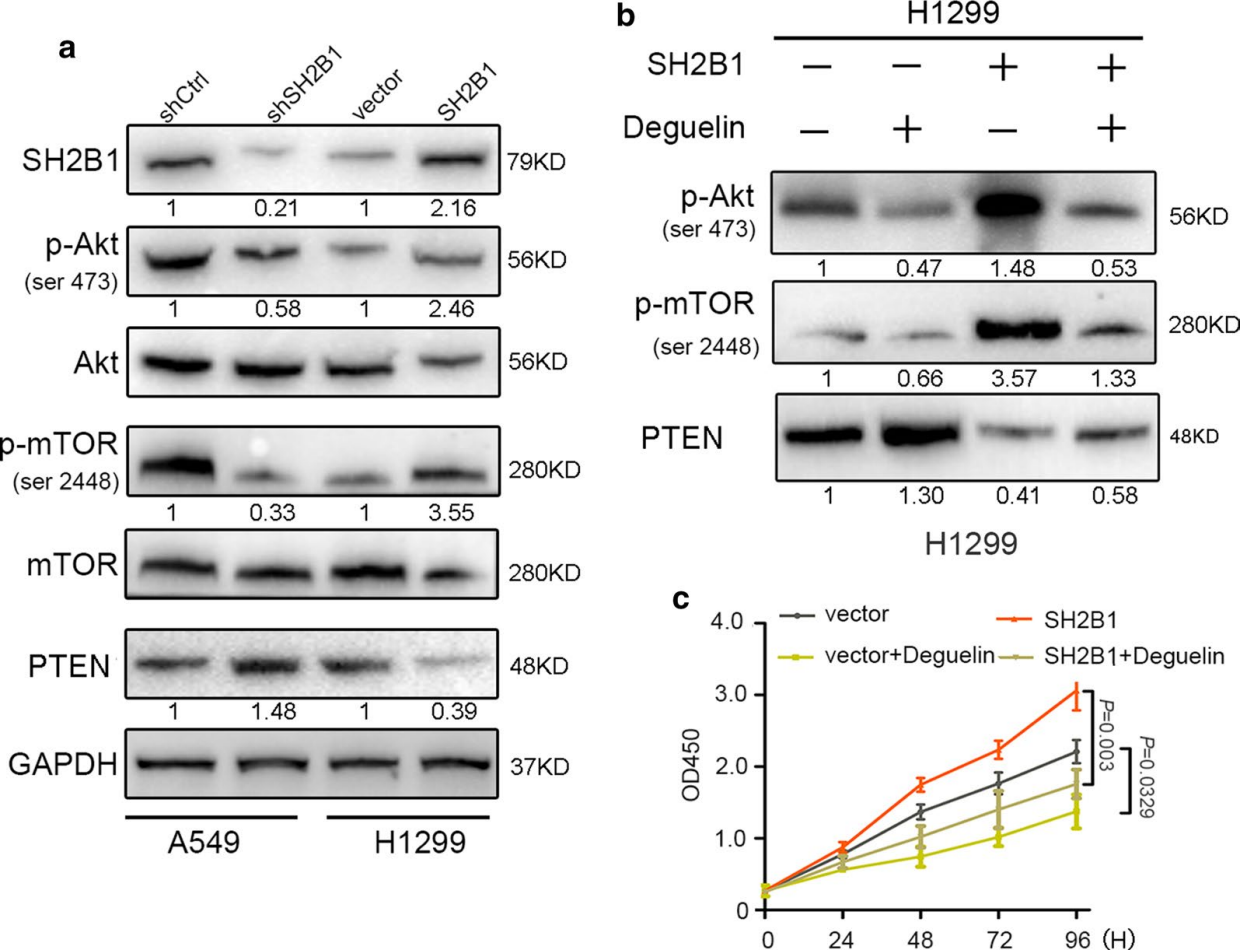
SH2B1 regulates NSCLC cell proliferation through Akt/mTOR pathway in vitro. **a**, **b** The effect of SH2B1 on Akt/mTOR signaling. **a** The protein levels of SH2B1, p-Akt, p-mTOR and PTEN in different panels of A549 and H1299 cells were assayed by Western blotting. **b** The protein levels of Akt/mTOR pathway targets (p-Akt, p-mTOR and PTEN) in different panels of H1299, which were subjected or not subjected to a Akt inhibitor, 10 μm/l deguelin for 24H, were assayed by Western blotting. Experiments were repeated three times and representative pictures are shown for **a** and **b**. GAPDH is used as an internal control. **c** CCK8 assay indicated that deguelin impaired the effect of SH2B1 on cell proliferation. Results are presented as mean ± SD

The above data indicated that SH2B1 was involved in the regulation of NSCLC cell proliferation. To elucidate the molecular mechanisms by which SH2B1 modulates NSCLC cell proliferation, we investigated the effects of knock-down and over-express SH2B1 on the phosphatidylinositol 3-kinase (PI3K)/Akt/mammalian target of Rapamycin (mTOR) signaling, which is an important regulator of cell proliferation and frequently aberrantly activated in human cancers [12, 13]. Western blot showed that transfection with SH2B1 shRNA significantly decreased Akt (Ser 473) and mTOR (Ser2448) phosphorylation, but increased PTEN expression in A549 cells (Fig. 4a). On the other hand, overexpression of SH2B1 induced activation of Akt and mTOR, but decreased PTEN expression in H1299 cells (Fig. 4a). Furthermore, we examined the effect of SH2B1 on Akt/mTOR signaling by using Akt specific inhibitor deguelin (Fig. 4b). We observed that the protein changes in Akt pathway targets (p-Akt, p-mTOR and PTEN) induced by SH2B1 over-expression reversed by deguelin (Fig. 4b). Importantly, NSCLC cells proliferation was increased in S2B1 over-expression cell (H1299-SH2B1), while the proliferation effect was dramatically impaired by deguelin (Fig. 4c) and mTORC1 inhibitor rapamycin (Additional file 1: Figure S2). Taken together, these data suggested that the Akt/mTOR signaling cascade might participate in SH2B1-induced cell proliferation in NSCLC cells.

### Clinical relevance of SH2B1 and its targets in NSCLC

**Fig. 5:**
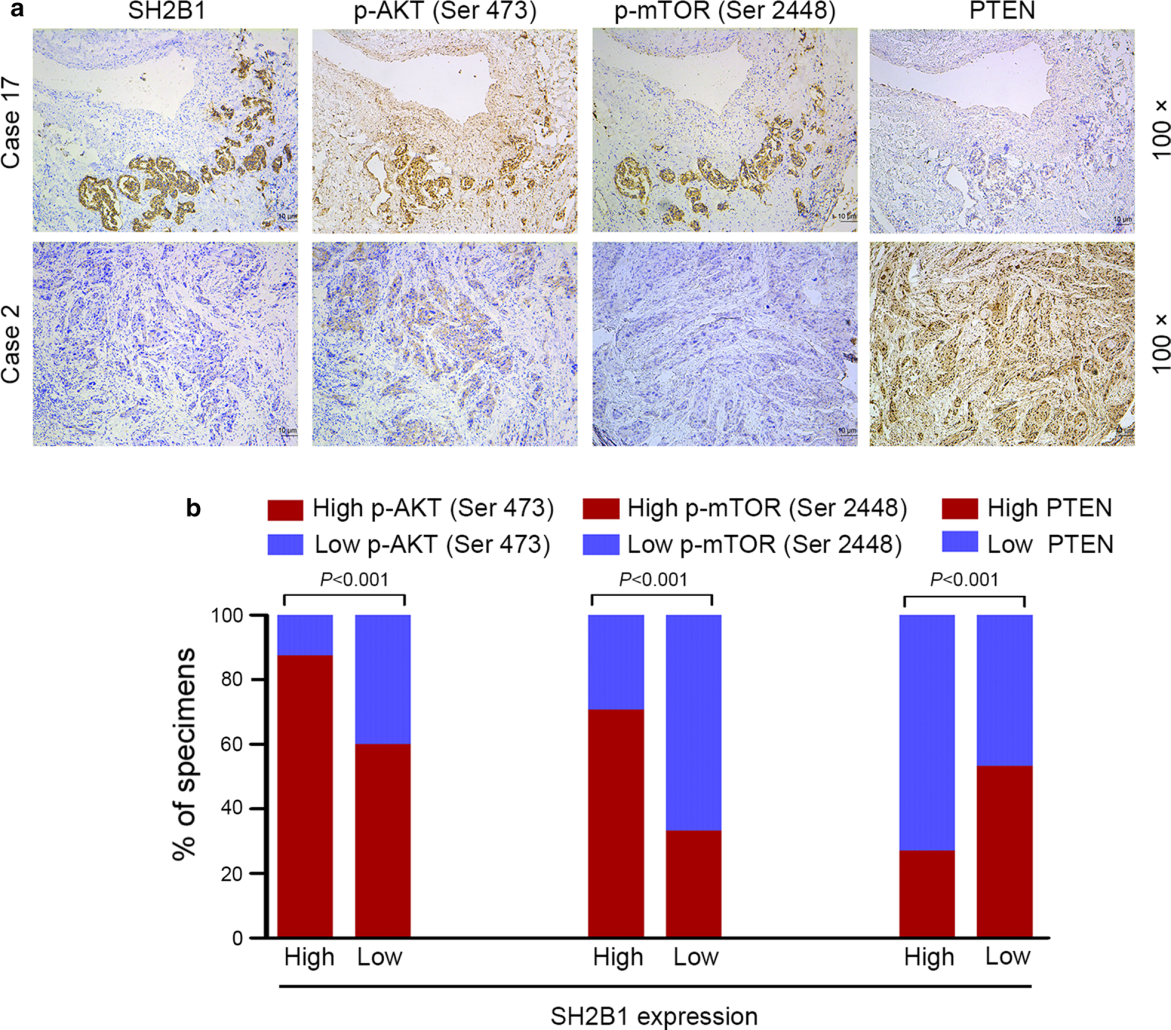
Clinical relevance of SH2B1 and its targets in NSCLC. **a** Representative expression levels of SH2B1, p-Akt (Ser 473), p-mTOR (Ser 2448) and PTEN in NSCLC by IHC staining. Scale bars, 10 μm. **b** Percentages of specimens showing high or low SH2B1 expression relative to levels of p-Akt (Ser 473), p-mTOR (Ser 2448) and PTEN (all *P *< 0.001)

Finally, we examined whether SH2B1 mediated Akt and mTOR activity, and PTEN suppression in NSCLC are clinically relevant. In 104 human NSCLC samples obtained from Xiangya hospital Thoracic Tissue Bank, IHC assay was performed. Representative pictures were depicted in Fig. 5a. IHC analysis revealed there was a significant correlation between SH2B1 expression and expression of p-AKT, p-mTOR and PTEN. In the Fig. 5b and Additional file 1: Figure S3, SH2B1 expression strongly correlated with p-Akt (Ser 473) and p-mTOR (Ser 2448) expression (*P *< 0.001), but inversely correlated with PTEN expression (*P *< 0.001). Collectively, these results further support the notion that SH2B1 overexpression activates Akt/mTOR signaling cascade leading to NSCLC progression.

## Discussion

In this study, we demonstrated the functional role of SH2B1 in NSCLC both in vivo and in vitro. Previous work showed that SH2B1, one of the SH2B adaptor protein family, mainly performed classical adaptor functions to recruit specific signal proteins [3]. In clinical disease, the research of SH2B1 mainly focused on energy imbalance, severe leptin resistance, obesity and type 2 diabetes [14, 15]. However, in cancer process, the adaptor protein SH2B1 is rarely well characterized. In this work, we identified a novel role of SH2B1 in modulation of cell proliferation in NSCLC.

It has been reported that SH2B1 was a key enhancer of proto-oncogene RET by interacting with proto-RET and RET/PTC associated onco-proteins to mediate RET-induced neoplastic transformation [9]. In addition, an emerging several basic research showed that SH2B1 was involved in mitogenic response [6], suggesting that SH2B1 could regulate cell proliferation by enhancing STATs signals [6]. Our precious study showed that the expression of SH2B1 was significantly higher in NSCLC tissues compared with their normal counterparts and was positively correlated with late-stages NSCLC patients and shorter survival time, which strongly indicated that SH2B1 may be a target molecule for blocking aberrant progression of NSCLC [4]. In the present study, we found that SH2B1 may play an important role in NSCLC cell cycle, apoptosis and proliferation. Firstly, knockdown of SH2B1 remarkably resulted G1 phase arrest in A549 cells, while overexpression of SH2B1 induced S phase arrest in H1299 cells which was further confirmed by enhanced expression of transcription factor c-Myc that is essential to cell proliferation and growth [16]. Secondly, knockdown of SH2B1 increased the number of apoptotic cells in A549 cells, which was further confirmed by enhanced expression of caspase 3, a key apoptosis-related factor [17]. Finally, knockdown of SH2B1 significantly impaired NSCLC cell growth, while overexpression of SH2B1 elevated cell growth in vitro and in vivo.

It has been well known from several studies that PI3K/Akt/mTOR signaling cascade is often aberrantly activated in a variety of cancer types and thus is an important regulator of cell proliferation and growth [18, 19]. And deregulation of phosphatase and tensin homologue (PTEN), which is well demonstrated to be a vital tumor suppressor and essential for prevention of tumorigenesis, has been identified to largely contribute to the development of cancers through constitutive termination of PI3K/Akt signaling [20, 21]. Our previous study has shown that systemic deletion of SH2B1 in generation of SH2B1-deficient mice inhibited the activation of PI3K/Akt and Erk1/2 pathways to develop age-dependent hyperinsulinemia, hyperglycemia and glucose intolerance [22], suggesting that SH2B1 is an upstream regulatory of PI3K/Akt signaling in endocrine disease. Here, we investigated the molecular mechanisms by which knockdown and overexpression of SH2B1 regulated NSCLC cell growth in vitro. Hence, we examined the effects of SH2B1 on Akt/mTOR/PTEN pathway. As expected, SH2B1 shRNA significantly decreased Akt (ser 473) and mTOR (ser 2448) phosphorylation in NSCLC A549 cells; however, no detectable changes in total Akt and mTOR protein were observed. Concordantly, overexpression of SH2B1 elevated p-Akt (ser 473) and p-mTOR (ser 2448) and decreased PTEN protein expression. Hence, overexpression of SH2B1 was shown to promote H1299 cells proliferation. Moreover, deguelin, an Akt inhibitor, significantly abolished SH2B1 overexpression-induced H1299 cells proliferation. In agreement with the results in vitro, using deguelin dramatically decreased the p-Akt and p-mTOR and increased PTEN expression in H1299 cells. Therefore, the enhanced NSCLC cell proliferation and growth in vitro and in vivo due to overexpression of SH2B1 could be explained, at least in part, by activation of Akt/mTOR pathway. However, the underlying mechanism for SH2B1 to activate Akt/mTOR/PTEN signaling cascade need to be further investigated.

In summary, our study revealed that SH2B1 overexpression had an important role in NSCLC progression and that SH2B1 was a critical activator of Akt/mTOR signaling. Understanding the role of SH2B1 in the pathogenesis of NSCLC and aberrant activation of Akt/mTOR signaling cascade may allow the development of new therapeutic target against NSCLC.

## Conclusions

Our findings indicated that SH2B1 played an essential role in NSCLC cell proliferation through promoting cell cycle and reducing apoptosis progression. The tumor-promoting effect of SH2B1 may be partially related the activations of Akt/mTOR/PTEN axis. In summary, our study provided a promising strategy for restraining tumor progression in NSCLC patients.

## Additional file

**Additional file 1: Figure S1.** Cell cycle analysis by propidium iodide (PI) staining showed that (A) SH2B1-knockdown slow down the S phase of A549 cell cycle progression (*P* = 0.0023, n = 3); (B) SH2B1 overexpression accelerated the S phase of H1299 cell cycle progression (*P* = 0.0110, n = 3). **Figure S2.** CCK8 assays were performed in H1299 cells with different treatment at 24 h, 48 h, 72 h, 96 h. Rapamycin impaired the effect of SH2B1 on cell proliferation (both *P* < 0.001). n = 3, bar: SD. **Figure S3.** Clinical relevance of SH2B1 and its targets in NSCLC. (A–C) Correlation between SH2B1 and expression of p-Akt, p-mTOR and PTEN with liner regression and Pearson’s significance. SH2B1 has a significant positive correlation with p-Akt (r = 0.614, *P* < 0.001), p-mTOR (r = 0.523, *P* < 0.001) expression and negative correlation with PTEN level (r = 0.406, *P* < 0.001) by IHC staining.

## Abbreviations

SH2B1: Src homology 2 (SH2) B adaptor protein 1
NSCLC: non-small cell lung cancer
TNM: tumor-nodule-metastasis
mTOR: mammalian target of Rapamycin
