# Supplementary material for: SH2B1 promotes NSCLC cell proliferation through PI3K/Akt/mTOR signaling cascade
Source: Cancer Cell Int. 2018 Sep 6;18:132. doi: 10.1186/s12935-018-0632-x (PMC6127928; doi:10.1186/s12935-018-0632-x)
Supplement: Supplementary file 1 — Additional file 1: Figure S1. Cell cycle analysis by propidium iodide (PI) staining showed that (A) SH2B1-knockdown slow down the S phase of A549 cell cycle progression (P = 0.0023, n = 3); (B) SH2B1 overexpression accelerated the S phase of H1299 cell cycle progression (P = 0.0110, n = 3). Figure S2. CCK8 assays were performed in H1299 cells with different treatment at 24 h, 48 h, 72 h, 96 h. Rapamycin impaired the effect of SH2B1 on cell proliferation (both P < 0.001). n = 3, bar: SD. Figure S3. Clinical relevance of SH2B1 and its targets in NSCLC. (A–C) Correlation between SH2B1 and expression of p-Akt, p-mTOR and PTEN with liner regression and Pearson’s significance. SH2B1 has a significant positive correlation with p-Akt (r = 0.614, P < 0.001), p-mTOR (r = 0.523, P < 0.001) expression and negative correlation with PTEN level (r = 0.406, P < 0.001) by IHC staining. [file 12935_2018_632_MOESM1_ESM.docx]

Additional file 1

**SH2B1 promotes NSCLC cell proliferation through PI3K/Akt/mTOR signaling cascade**

Shaoqiang Wang^1^, Yingying Zheng^2^, Zhiwei He^3^, Wolong Zhou^3^, Yuanda Cheng^3#^ & Chunfang Zhang^3#^


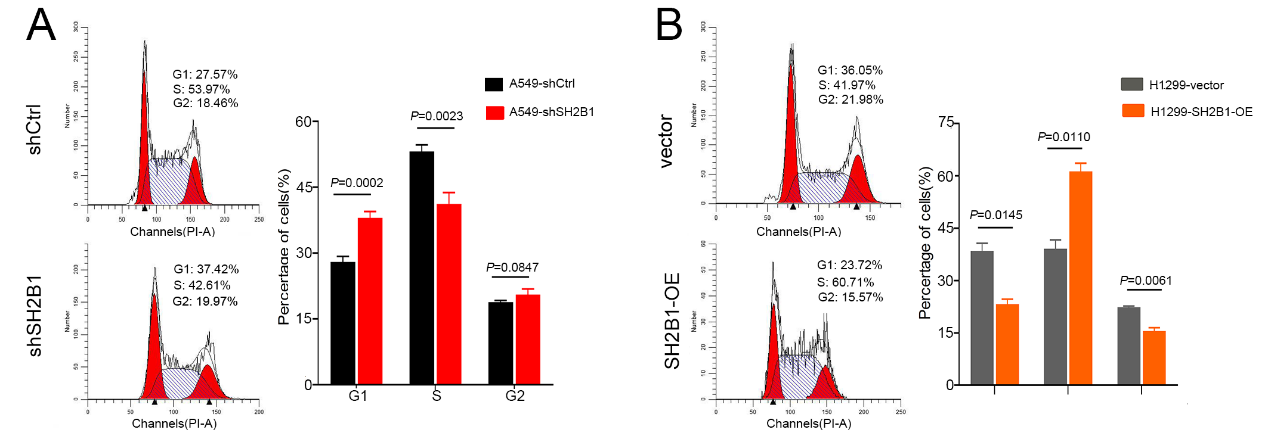


Figure S1. Cell cycle analysis by propidium iodide (PI) staining showed that (A) SH2B1-knockdown slow down the S phase of A549 cell cycle progression (*P*=0.0023, n=3); (B) SH2B1 overexpression accelerated the S phase of H1299 cell cycle progression (*P*=0.0110, n=3).





Figure S2. CCK8 assays were performed in H1299 cells with different treatment at 24h, 48h, 72h, 96h. Rapamycin impaired the effect of SH2B1 on cell proliferation (both *P*<0.001). n=3, bar: SD.


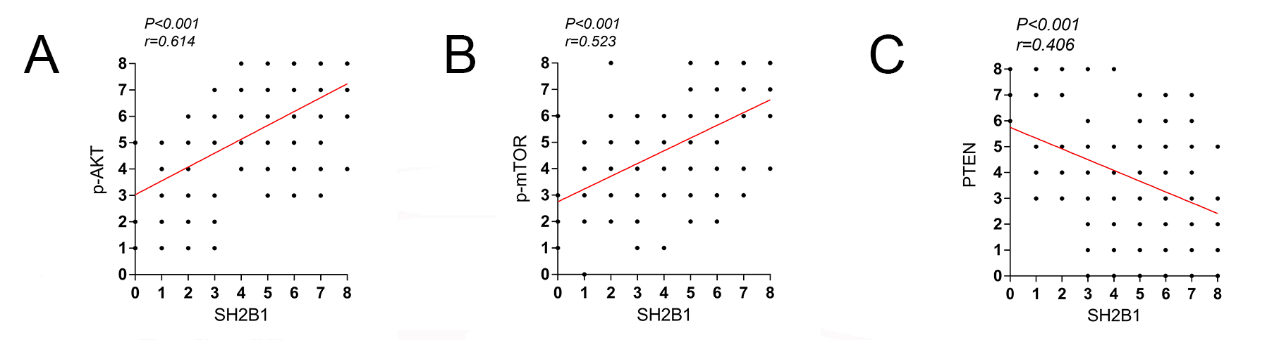


Figure S3. Clinical relevance of SH2B1 and its targets in NSCLC. (A-C) Correlation between SH2B1 and expression of p-Akt, p-mTOR and PTEN with liner regression and Pearson's significance. SH2B1 has a significant positive correlation with p-Akt (r=0.614, *P*<0.001), p-mTOR (r=0.523, *P*<0.001) expression and negative correlation with PTEN level (r=0.406, *P* < 0.001) by IHC staining.
